# Effects of Sudden Changes of Temperature

**Published:** 1847-09

**Authors:** 


*Effects of sudden changes of Temperature.*—It is not unusual," says Von Littrow, (*Miscellaneous Writings*, Edited by his son: Stuttgart, 1846,) for sitting rooms [in Russia] to be heated to 30° Reaumur (99° Fahrenheit), whilst the temperature out of doors is as many degress below zero. Now, as the inmates of such rooms must leave them more or less every day, and expose themselves to the open air, they must undergo a sudden change of temperature of fully 60° (135° Fahrenheit), which not even a Russian constitution could endure, unless peculiar precautions were taken. The middle-aged foreigner who settles in these regions, and chooses to adhere to the customs of his own warmer country, generally succumbs quickly to the severity of the climate. In Perm, for instance, a town that is south of the latitude of St. Petersburgh, I was told by a young German, who had been settled there for six years, that not one of his countrymen, whom he had found there on his arrival, still survived. 'Within these six years,' he said 'I have followed more than twenty German fathers of families to the grave, and I should probably myself have had a like fate, if I had not arrived here in my twenty-third year, at an age when the constitution is sufficiently plastic to accommodate itself to new outward circumstances.—The Russians,' he added, 'know this veiy well, and they look on every foreigner who comes among them in his fortieth year, as certainly destined to die soon. Thus, the parents all die premature, but their children in genral thrive very well.' The frequent and sudden exchange of a hot room for the cold outer air produces a malady peculiar to those northern regions, and which is the more appaling since it must be remedied on the instant, otherwise it will be rapidly fatal, or will end in a very distressing chronic malady. The strongest and healthiest man, if he put one foot out of the room, or if the door or window is opened for a moment, is often seized with an uneasy sensation, which is immediately followed by an extreme disturbance of his whole system, the consequence of the sudden suppression of perspiration. A great weariness in the limbs, a feeling in the extremities as if they would drop off, piercing headache, and a burning in the eyes, are the first somptoms of the disorder, and if they are not immediately remedied, the case is soon beyond curing. The grand requisite is to restore the suppressed perspiration. To this end the invalid is put into bed without delay, with his clothes on, heaps of blankets and furs are laid over him, and he is made to drink as much very hot tea as he can swallow. The patient has no sooner gulped this down, and drawn in his head under the clothes, than a copious perspiration breaks out over his whole body, and all the alarming symptoms vanish as rapidly as they first appeared. The rest of the company, who have meanwhile seated themselves again round the table, are not at all surprised to find the sick man sticking his head out from under his mountain of furs in the next quarter of an hour, and chatting with them as gaily as if nothing had happened, whereas to any one not familiar with such cases, he would have seemed a few minutes before but a lost man. The coverings are gradually taken off, and the patient is often quite well again the same evening, and as hearty as ever.

But the case is very different with those who are not thus relieved on the instant. If they are not dead by the next day, which most commonly happens, they remain crippled in every joint, and die a painful, lingering death. These people may at once be recognised, not only by their crippled limbs, but by a peculiar cachectic expression of countenance. Their answer, when asked what is the matter with them, is—*Prosdudilsa*, 'I have had a chill,' a word that smites with as awful a sound on the Russian ear as ever *danatos* did on that of a Greek of old.

Whoever is not capable of being instantly thrown into a copious perspiration by a few cups of hot drink, will, if he takes my advice, keep away from those regions. But how is it that there are no such unfortunate persons among the Russians? I never met with any. Just as many persons can fall asleep whenever they like, so all Russisns can perspire at will. Give them a cup of tea, a warm cloak, and thick cap, and the thing is done. They may thank themselves for this precious peculiarity, for such it really is. Their frequent use of hot baths keeps the pores of their skin open, and their copious draughts of warm tea increase the excreting power of the skin, adapt it to resist the influences of their climate,—influences which, but for these counteracting causes, would perhaps be more pernicious to the population of Russia than even the plague is to the people of the East.—*Westminster and Foreign Review.*

